# Toxicity Reduction in the Treatment of HPV Positive Oropharyngeal Cancer: Emerging Combined Modality Approaches

**DOI:** 10.3389/fonc.2018.00439

**Published:** 2018-10-09

**Authors:** Sarah Deschuymer, Hisham Mehanna, Sandra Nuyts

**Affiliations:** ^1^Department of Radiation Oncology, KU Leuven, University Hospitals Leuven, Leuven, Belgium; ^2^Institute of Head and Neck Studies and Education, University of Birmingham, Birmingham, United Kingdom

**Keywords:** head and neck cancer, oropharyngeal cancer, human papillomavirus, HPV, de-intensification trials

## Abstract

Human papillomavirus positive (HPV+) oropharyngeal squamous cell carcinoma (OPC) is a distinct clinical entity within the head and neck cancers, with a unique epidemiology and, in general, a favorable prognosis. Because of this favorable prognosis, researchers have considered de-intensifying the current standard treatment of HPV+ OPC in order to reduce acute and late treatment related toxicity without compromising outcome. Current ongoing trials can be divided in three main categories: de-intensification of the chemotherapy by replacing concomitant platinum-based chemotherapy with the EGFR-inhibitor cetuximab, or de-intensification of the radiation dose of either the primary radiotherapy of selected, good-responding patients after induction chemotherapy or of the adjuvant radiotherapy based on pathology features after primary surgery. Despite the good prognosis of the majority of HPV+ OPC patients, a proportion of them still have poor prognosis. This unmet need has led clinical research on new treatment strategies focused on influencing the unique micro-environment of HPV+ OPC with for example immunotherapy. This article summarizes the current understanding regarding the optimal treatment of non-metastatic HPV+ OPC. Ongoing and published clinical trials regarding de-intensification strategies, immunotherapy and proton therapy are described focusing on the rationale and underlying evidence of these emerging treatment strategies. Nevertheless, until the results of the ongoing trials are known, the treatment of HPV+ OPC in clinical practice should remain identical to the treatment of HPV negative OPC.

## Introduction

Oropharyngeal squamous cell carcinomas (OPC) are tumors located in the soft palate, the pharyngeal wall, the tonsils or the base of tongue, the latter two being the preferred location of Human Papillomavirus related (HPV+) OPC. The incidence of OPC is increasing in the developed countries, chiefly attributed to the epidemic increase in incidence of HPV+ OPC (1, 2).

HPV+ OPC has a better prognosis than tobacco and alcohol related (HPV–) OPC. They should, therefore, be considered as two distinct clinical entities. This is reflected in the new AJCC/UICC TNM 8th edition (8th Ed) staging system with a different classification for HPV+ and HPV– OPC (3). The new clinical (c) TNM 8th Ed for HPV+ OPC contains adjustments in both T- and N-classification. cT-classification remained unchanged except for the disappearance of the distinction of the T4-classification in T4a and T4b. The cN-classification, on the other hand, has changed extensively: N0 is the absence of malignant lymph nodes, N1 is reserved for one or more ipsilateral lymph nodes smaller than 6 cm, N2 is the presence of contralateral or bilateral lymph nodes smaller than 6 cm while N3 is one or more lymph nodes larger than 6 cm. The presence of extranodal extension is not a classification parameter in contrast to the TNM 8th Ed for HPV– OPC. Table 1 shows the differences in the clinical group staging between the 7th and 8th Ed for HPV+ OPC. In the pathological (p) TNM 8th Ed classification, pT-classification is the same as cT-classification while pN-classification is exclusively defined by the number of pathological lymph nodes.

**Table 1:** Differences in clinical group staging between the 7th and 8th edition AJCC/UICC TNM classification system (cTNM) for Human Papillomavirus related oropharyngeal squamous cell carcinoma.

**Stage**	**TNM 7th edition**	**cTNM 8th edition**
I	T1N0	T1T2-N0N1
II	T2N0	T3-N0N2; T1T2-N2
III	T3-N0N1; T1T2-N1	T4N_any_; TxN3
IV	IVa: T4a-N0N2c; T1T3-N0N2c IVb: T4bN_any_; T_any_ N3 IVc: T_any_N_any_M1	T_any_N_any_M1

Although the prognosis of HPV+ OPC is better than that of HPV– OPC, currently, the treatment of these two entities is identical (4). Nevertheless, researchers have attempted to de-intensify the treatment of HPV+ OPC to minimize treatment related toxicity without compromising the oncologic outcome. On the other hand, a part of the HPV+ OPC still have poor prognosis directing clinical research to new treatment strategies focusing on influencing the unique micro-environment of HPV+ OPC with for example immunotherapy. In this paper, we will discuss the current treatment of HPV+ OPC, the ongoing or completed de-intensification trials, their results and underlying rationale. Last, we will briefly describe the potential place of immunotherapy and proton therapy in HPV+ OPC. The review was based on a literature search of PubMed with the Medical Subject Heading term “oropharyngeal cancer” AND “human papillomavirus” combined with the key words “radiotherapy,” “toxicity,” “de-escalation,” “de-intensification,” and “dose reduction.” The PubMed search was combined with back tracking based on published reference lists.

## Current treatment

The treatment of HPV+ OPC depends on patient related characteristics in combination with tumor location, tumor extension, lymph node status and relies, as a result, on accurate staging. The staging and treatment of HPV+ OPC and more generally of head and neck squamous cell carcinoma (HNSCC) can generally be divided in two categories, early vs. locally advanced disease.

Early disease, (T1 or T2 tumor with maximum one ipsilateral malignant lymph node smaller than 3 cm), is treated with a single modality treatment, surgery or radiotherapy (RT). Locally advanced disease is treated with combined modality treatment consisting of either RT with concomitant chemotherapy (CRT) or cetuximab or of surgery followed by adjuvant RT or by adjuvant CRT in case of positive resection margins or extranodal extension (ENE) (5–9). Treatment decisions are made by a multidisciplinary setting, and take into account patient characteristics and the anticipated functional outcomes after surgery.

The added value of concomitant platinum-based chemotherapy in addition of primary RT treatment of locally advanced disease has been demonstrated in a large meta-analysis of 9615 subjects (5). Trials with addition of induction chemotherapy (ICT) to CRT have failed to demonstrate any benefit in overall survival or progression free survival and ICT is therefore not considered standard-of-care (5, 10, 11). Alternatively, the addition of cetuximab, a chimeric epidermal growth factor receptor (EGFR)-inhibitor, in combination with primary radiotherapy has shown improved overall survival, but only in one study including 424 patients (6). Since the two different concomitant systemic therapies, platinum-based chemotherapy and cetuximab, in addition to RT were never compared head-to-head in a randomized controlled trial and the evidence for the use of platinum-based chemotherapy is based on a larger dataset, RT plus concomitant platinum-based chemotherapy is favored, while cetuximab can be given to patients with contra-indications for platinum derivates.

## Changes in primary (chemo)radiotherapy

Radiotherapy induces treatment related toxicities correlated to the RT dose delivered to normal tissues (12, 13). Moreover, concomitant systemic treatment significantly increases the acute and late toxicity (6, 14). This toxicity strongly influences the quality of life of cancer patients (15). The avoidance or diminution of treatment related toxicity becomes more prominent in patients with a good long-term prognosis, such as in HPV+ OPC. For this reason, researchers have attempted to reduce toxicity by changing or leaving out the concomitant therapy or by reducing the RT dose. First, we will discuss the changes in the concomitant systemic therapy. Next, we will discuss the trials with reduced RT dose and with RT dose adaptation after ICT.

### Replacement of cisplatin by cetuximab

Cisplatin increases the acute and late toxicity with severe mucositis, dermatitis, dysphagia and potential life threatening neutropenic fever, while the use of cetuximab is classically only associated with the typical acneiform rash, hypomagnesemia and infusion reaction (5–7, 14, 16). In addition, a subgroup analysis of the Bonner trial, although underpowered and unplanned, showed that especially younger patients with oropharyngeal cancer, early T stage and advanced N-stage had an improved overall survival with cetuximab-RT compared to RT only (6). The hypothesis rose that these patients for whom cetuximab treatment would be the most beneficial, were HPV+ OPC, typically presenting at younger age with small primary tumors and multiple lymph nodes.

Several running de-intensification trials hypothesize that treatment with cetuximab-RT is non-inferior to CRT for HPV+ OPC and that cetuximab is associated with a more favorable treatment related toxicity profile and better long-term quality of life. The De-ESCALaTE trial (NCT01874171) and TROG 12.01 trial (NCT01855451) compare the toxicity of both treatments, whereas the largest trial, the RTOG 1016 (NCT01302834), including around 1,000 patients, is currently the only randomized controlled trial with oncologic outcome as primary endpoint (Table 2). Although this treatment approach is promising, the efficacy of cetuximab in HPV+ OPC is controversial. Several researchers are convinced that only HPV– OPC can benefit from cetuximab based on several studies demonstrating an inverse relationship between EGFR expression and detection of HPV. In addition, the Cancer Genome Atlas group, examining at the cumulative effect of various mechanisms of biological alterations in HNSCC, suggested EGFR as a relevant oncogenic target but only in HPV– OPC (17–20). In contrast, Rosenthal et al. conducted a retrospective subset analysis of the IMCL-9815 trial of Bonner et al. focusing on the potential impact of p16 status (a surrogate marker of HPV positivity) on the outcome of 182 OPC patients (6, 21). They showed benefit for cetuximab on locoregional control and overall survival in both p16+ and p16– subgroup. Although their data suggested a more pronounced gain from cetuximab in the p16+ subgroup, no significant interaction between treatment group and p16 status was shown, confirming p16 status as a prognostic biomarker, though not a predictive biomarker (21). Interestingly, EGFR expression is also a prognostic biomarker but not predictive for the efficacy of cetuximab (22). Many now believe that the antitumoral activity of cetuximab is mainly an immunologic response on the non-human part of the antibody by potentiating the cytotoxic T-cell antitumor immune response, rather than through EGFR-inhibition (23, 24). This could explain why trials with fully human EGFR-inhibitors, like panitumumab, have failed to demonstrate any survival benefit compared to or in addition to platinum-based chemotherapy (25,–28). HPV+ OPC could potentially benefit more of the enhanced immune response by cetuximab than HPV– OPC since HPV+ OPC contains elevated T- and B-lymphocyte infiltration and expresses viral proteins (24). To conclude, despite considerable research devoted to this topic, many questions with respect to the use of EGFR-inhibitors and in particular of cetuximab remain unanswered until now. Results of the afore-mentioned trials will hopefully bring clarification. Interestingly, an interim analysis of the RTOG 1016 trial found that treatment with RT and cetuximab is associated with worse overall and progression-free survival compared to the current standard treatment with RT and cisplatin (29).

**Table 2:** De-intensification trials replacing cisplatin by cetuximab.

**Name study**	**Design**	**Inclusion TNM 7th**	**Inclusion TNM 8th**	**Smoking**	**Primary endpoint**
De-ESCALaTE NCT01874171	70 Gy + 3-weekly cddp vs. 70 Gy + weekly Cetuximab	T3T4-N0; T1N1-T4N3	T3T4-N0; T1N1-T4N3	Exclusion if more than 10 PY and more than one ipsilateral LN, contralateral LN or LN > 6 cm	Acute and late toxicity (2Y)
TROG 12.01 NCT01855451	70Gy + weekly cddp vs. 70 Gy + weekly Cetuximab	T3-N0N2c; T1T2-N2aN2c	T3-N0N2; T1T2-N1N2 (excluding N1 with only one ipsilateral LN < 3 cm)	Exclusion if more than 10 PY and more than one ipsilateral LN or contralateral LN	Symptom severity: acute toxicity
RTOG 1016 NCT01302834	70 Gy + 3-weekly cddp vs. 70 Gy + weekly Cetuximab	T1T2-N2aN3; T3T4-N_any_	T1T2-N1N2 (excluding N1 with only one ipsilateral LN < 3 cm); T3T4-N_any_	/	Overall survival
NCT01663259	One arm: 70 Gy + weekly cetuximab	T3-N0N2c; T1T2-N1N2c	T3-N0N2; T1T2-N1N2	< 10 PY	Recurrence rate (3Y)

*cddp, cisplatin; LN, Lymph nodes; PY, smoking pack years; 2Y,3Y, up to 2 or 3 years after end of treatment*.

Beside treatment efficacy, we must consider other potential pitfalls of these de-intensification trials. The TROG 12.01 trial compares the acute toxicity of radiotherapy (70 Gy) plus weekly cisplatin with radiotherapy (70 Gy) plus weekly cetuximab. A recent systematic review and meta-analysis of Szturz et al. compared two different cisplatin schedules, the traditional 3 weekly high-dose vs. the weekly low-dose regimen, in combination with altered radiotherapy and demonstrated less complications in terms of severe acute mucositis, constipation, toxic deaths and severe late subcutaneous fibrosis in patients receiving the 3 weekly high-dose cisplatin regimen. In addition, the overall survival and compliance differed significantly in favor of the 3-weekly schedule (30). The potential observed toxicity differences in the TROG 12.01 could therefore be not representative of a 3-weekly cisplatin regimen. Furthermore, the toxicity results of the TROG 12.01 trial will be difficult to compare with the results of the De-ESCALaTE trial, examining the toxicity of radiotherapy (70Gy) plus 3-weekly cisplatin vs. radiotherapy (70Gy) plus weekly cetuximab, as their control arms may have a different toxicity profile.

Another concern is the wide inclusion criteria of the RTOG 1016 trial, including T1T2-N2aN3 and T3T4-N_any_ (TNM 7th Ed) and the influence on the distant metastasis rate. Although the MACH-NC group did not demonstrate an influence of concomitant chemotherapy on distant metastasis, O'Sullivan et al. have shown that the benefit of cisplatin on distant metastasis in N0N2a disease is limited, while in N2cN3 disease and in heavy smokers with N2b-disease (7th Ed) chemotherapy has a significant effect (5, 31). In contrast, the Bonner trial has shown improvement by cetuximab of the locoregional control, progression free survival and overall survival but has failed to show an effect on distant metastasis (6). The replacement of cisplatin by cetuximab in N2b heavy smokers or N2c-N3 disease could have detrimental effects on the development of distant metastasis and by consequence on the overall survival. It will be important to keep in mind the O'Sullivan et al. study when interpreting the results of the RTOG 1016 trial.

Lastly, the study design of the RTOG 1016 trial, namely a non-inferiority trial, holds some disadvantages. In non-inferiority trials, minor differences are accepted and demonstration of non-inferiority is therefore not the demonstration of equivalence. A sufficient number of deaths must happen to provide enough statistical power for analysis otherwise potential inferiority might not be ruled out due to wide confidence intervals. Before the start of the trial, the researchers must carefully select the minimum clinically relevant difference, commonly called delta. This delta must be substantially smaller than the estimated benefit of the active treatment, cisplatin, otherwise it could happen that the new treatment, cetuximab, is not better than placebo but gets accepted as non-inferior (32, 33). Interestingly, Brotherston et al. conducted an investigation with questionnaires assessing patients' preferences regarding the acceptable delta for de-intensification cancer treatment. They showed that patients' primary concern was survival with 35% of the patients unwilling to risk any drop in survival probability, even if it implied less treatment related toxicity, and a further 34% of patients willing to accept maximum 5% reduction in survival probability (34). We must therefore be cognizant that the priorities of patients might be different that those of researchers.

### Radiotherapy dose reduction

HPV+ OPC is believed to be more radiosensitive than the HPV– OPC and may be cured with doses less than 70 Gy (35). A lower RT dose delivered to the tumor might lead to a lower dose on the surrounding normal tissue and to less toxicity with the same good oncologic outcome. This was first investigated in a prospective, multi-institutional, phase II study in which all patients were treated with RT at 60 Gy at 2Gy per fraction, 5 days a week with weekly low-dose cisplatin, 30 mg/m2 (Table 3). Four to eight weeks after completion of RT, all patients were evaluated for clinical complete response (cCR), defined as no measurable tumor present on physical and radiologic examination, followed by planned surgical evaluation to asses pathologic complete response (pCR). In patients who had a cCR at the primary site, directed biopsies of the primary site were taken while minimally invasive resection was performed if there was no cCR at the primary site. All patients who had node-positive disease before RT had selective nodal dissection. The pCR rate at the primary site was 86% and in the neck 98% (36). Recently, the long-term follow-up was published with an observed 3 year cause-specific survival of 100% and an OS rate of 95% (37). It is, however, not possible to determine if the planned surgical evaluation was therapeutic because the clonogenic viability of the residual foci could not be determined by microscopic examination. CRT followed by surgery in all patients is probably an overtreatment and an unnecessary enhancement of toxicity, although in this trial the patients' reported long term symptom burden was low to moderate. Patient selection is opportune and is under investigation in the follow-up study (NCT02281955). Patients will receive the same de-intensified CRT regimen, followed by a 12-week post-CRT positron emission tomography/CT to guide the use of surgery (36–38).

**Table 3:** De-intensification trials with reduced RT dose.

**Name study**	**Design**	**Inclusion TNM 7th**	**Inclusion TNM 8th**	**Smoking**	**Primary endpoint**
NCT01716195	One arm: ICT (2 cycli paclitaxel-carboplatin) + response adapted RT (54 Gy or 60 Gy) with weekly paclitaxel	T1T2-N2aN3; T3T4-N_any_	T1T2-N1N2 (excluding N1 with only one ipsilateral LN < 3 cm); T3T4-N_any_	/	2Y PFS
NCT01530997	One arm: 60Gy + weekly cisplatin	T0T3-N0N2c	T1T3-N0N2	< 10PY or < 30PY and abstinent >5Y	Pathologic Complete Remission
NRG HN002 NCT02254278	Reduced 60 Gy + weekly cisplatin vs. 60 Gy	T1T2-N1N2b; T3-N0N2b	T1T2-N1; T3-N0N1	< 10 PY	2Y PFS grade 3 dysphagia
ECOG 1308 NCT01084083	ICT (3 cycli of cisplatin, paclitaxel, cetuximab), then response adapted RT (54 or 69.3 Gy) with cetuximab	Resectable disease T3T4-N0; T1N1-T4N3	Resectable disease T3T4-N0; T1N1-T4N3	/	2Y PFS
The Quarterback Trial NCT01706939	ICT (TPF), patients with CR/PR randomized between RT (56Gy) with carboplatin vs. RT (70Gy) with carboplatin	T3T4-N0; T1N1-T4N3 OPC/CUP/nasopharynx	T3T4-N0; T1N1-T4N3 OPC/CUP/nasopharynx	Exclusion of active smokers or >20 PY	3Y PFS

*ICT, induction chemotherapy; TPF, docetaxel, cisplatin, 5-fluorouracil; CR/PR, complete response / partial response; RT, radiotherapy; OPC, Oropharyngeal carcinoma; CUP, carcinoma of unknown primary; PY, smoking pack years; 2Y, up to 2 years after end of treatment; PFS, progression free survival*.

Another research group has de-intensified the treatment even further by eliminating the concomitant therapy completely in combination with lowering the RT dose. The NRG HN002 trial (NCT02254278) randomized patients between RT dose of 60 Gy, one fraction a day for 6 weeks, with or without weekly cisplatin. Their inclusion criteria are based on the research of O'Sullivan et al. showing equal effect in terms of distant metastasis of RT, mostly accelerated regimens, and CRT for N0-N2a and N2b disease with less than 10 pack years (31). Notably, the RT regimen of the HN002 trial is significantly different from the regimen of the trial of O'Sullivan. This NRG HN002 trial is set up with a conventional fractionation regimen up to 60 Gy instead of the standard RT dose of 70 Gy or the accelerated RT regimen from the study of O'Sullivan et al. meaning this trial consists of two nonstandard arms. Even more, the time till the primary endpoint, 2 year progression free survival, might be too short to measure the effect of leaving out the concomitant therapy. Several publications have shown that the distant metastasis rate of HPV+ and HPV– OPC is similar but the timing of onset is different with the curve of HPV+ OPC continuing to increase for up to 5 years after treatment in contrast to the rather stable curve of HPV– OPC beyond 2 years (31, 39).

### Dose adaptation after induction chemotherapy

A meta-analysis of five randomized trials including over 1,000 patients could not show an OS or PFS benefit of induction chemotherapy (ICT) with docetaxel, cisplatin and 5-FU (TPF) compared to definitive CRT without induction chemotherapy in locally advanced HNSCC (10). The Eastern Cooperative Oncology Group (ECOG) published in 2007 a phase II trial (E2399) of taxane-based induction chemotherapy followed by CRT and obtained high organ preservation rate with low toxicity for OPC (40). Based on these results the ECOG investigated in a phase II trial, E1308, the further use of ICT. The purpose of the ICT was not to improve OS, but to reduce the tumor burden to subclinical disease in patients with HPV+ OPC and to allow in good responders the use of a reduced RT dose, 54 Gy instead of 70 Gy, to eradicate the residual lower tumor burden (40, 41). This lower RT dose to the tumor might lead to lower doses on the surrounding normal tissue and subsequently to less treatment related toxicity, such as dysphagia, feeding tube dependency, and better post-treatment quality of life. Cisplatin, as concurrent chemotherapy, was in this trial also replaced by cetuximab so the same concerns about the efficacy of cetuximab in HPV+ OPC as described above arise.

There is of course the concern that in patients who do not have a complete response after ICT, ICT will not improve the survival but will delay the start of the potentially curative RT treatment of the radiosensitive HPV+ OPC. In another phase II trial (NCT01716195) with dose adaptation after ICT, 2 cycles of paclitaxel and carboplatin, complete or partial responders received RT 54 Gy with weekly paclitaxel, while less than partial or no responders received RT 60 Gy with weekly paclitaxel. Although all patients in this trial received a lower RT dose than the standard 70 Gy, this treatment approach was associated with a high 2y progression-free survival of 92% (42).

The results of the E1308 trial were published in 2017 showing a high rate of clinical complete response after ICT (70%) with excellent 2y-OS of 94% and good toxicity profile according to the authors. The published acute treatment related toxicity is however worth mentioning, with 2 out of 80 patients only receiving one out of 3 cycles of ICT due to grade 3 or more toxicity. Fourteen patients had dose adaptations of cisplatin during ICT due to grade 3 or more hematologic toxicity, neuropathy or tinnitus and 18 patients had dose modification of cetuximab due to grade 3 or more acneiform rash, mucositis or hypomagnesemia. It is debatable if the reduction of RT dose and of the RT-related toxicity really outweigh the added toxicity of ICT. ICT with TPF was associated with 6.6% treatment-related toxicity in the recently published GORTEC 2007-02 phase III trial randomizing HNSCC patients between RT 70 Gy with carboplatin-5FU vs. 3 cycles of TPF followed by cetuximab-RT 70 Gy (43). The Quarterback trial (NCT01706939), another phase III dose reduction trial after ICT, randomizes patients with good response after ICT between 56 and 70 Gy concomitant with carboplatin. It should be noticed that this trial also includes, besides HPV+ OPC, nasopharyngeal cancers and cancers of unknown primary with p16+ squamous cell carcinoma histology (Table 3). To our knowledge the prognostic value of p16+ in HNSCC subsites other than OPC is not proven and trials should therefore only include HPV+ OPC patients to avoid bias and under-treatment of the other tumor subsites.

## Changes in primary surgery ± adaptive (C)RT

Surgery with or without adjuvant RT or CRT is an alternative treatment strategy in HPV+ OPC if the anticipated functional outcome after surgery is acceptable. Retrospective data have shown similar oncologic outcome between open surgery and radiotherapy. However, the rate of severe complications in the surgery group was higher (44). It should be pointed out that the surgical landscape has changed drastically since this published comparison. Minimal invasive surgery such as the transoral laser approach (TLM) or the transoral robotic surgery (TORS), have gained prominence in the last decade. These techniques, when performed by trained surgeons, provide similar oncologic outcome as the classic approaches, while avoiding mandibulotomy (45). As a result, they are associated with fewer complications and functional deficits compared to the classic approaches with mandibular split. To date, a prospective randomized clinical trial concerning oncologic and functional outcome of minimal invasive surgery vs. CRT has not yet been published. Interestingly, two randomized ongoing trials, the “Best of” EORTC 1420 trial (NCT02984410) and the ORATOR trial (NCT01590355), will compare the treatment related toxicity of TORS and RT or CRT (46, 47).

The extent of benefit from adjuvant treatment after surgery is based on the pathology following resection. Currently, it is unclear if the decision for postoperative (C)RT and the RT dose in HPV+ OPC must be based on the same pathology features as in HPV– OPC. In HPV– OPC, ENE is considered a negative prognostic factor and is now incorporated in the most recent clinical and pathologic nodal staging classification of TNM 8th Ed. In contrast, ENE was not adopted in the clinical nor in the pathological TNM 8th edition for HPV+ OPC, even though a recent analysis from the American national cancer data base, including over 1,000 HPV+ OPC who underwent primary surgery with negative resection margins, showed that ENE was an independent risk factor for worse prognosis in patients with HPV+ OPC. Surprisingly, adjuvant CRT compared with RT was not associated with a better OS in this population (48).

At the moment, there are three de-intensification trials trying to determine the optimal adjuvant treatment for HPV+ OPC after minimal invasive surgery. The ECOG 3311 trial (NCT01898494) tries to determine the optimal RT dose by dividing patients in three risk groups after TORS. The low-risk group without adverse pathology features does not receive adjuvant treatment. The intermediate risk group patients with clear margins, < 1 mm ECE, 2-3 positive lymph nodes, perineural invasion or lymphovascular invasion is randomized between RT up to 50 Gy or to 60 Gy. The high risk group with positive margins or >1 mm ECE or ≥4 positive lymph nodes receive standard CRT. The primary endpoint of this trial is 2-year progression free survival. The PATHOS trial (NC02215265) will, in addition, investigate the benefit of concomitant chemotherapy in the high risk group. Patients with positive margins or ECE are randomized between RT 60 Gy with or without concomitant chemotherapy. The ADEPT study (NCT01687413) only focuses on the benefit of chemotherapy in patients with ECE and negative margins and randomizes them between RT 60 Gy with or without concomitant chemotherapy.

## Other emerging treatment strategies

### Immunotherapy

Since the results of the CheckMate-141 study were published, immunotherapy has become standard of care in recurrent or metastatic HNSCC after platinum-based chemotherapy. The OS benefit of nivolumab, a PD-1 monoclonal antibody, was independent of p16 status, although the benefit was more pronounced in the p16+ OPC (49). The Keynote-012 study which investigated the efficacy of a similar PD-1 antibody, pembrolizumab, also observed a higher response to pembrolizumab in patients with recurrent or metastatic HPV+ OPC vs. recurrent or metastatic HPV– HNSCC (50, 51). The role of radiotherapy and the synergy with immunotherapy as adjuvant or concomitant treatment for advanced HPV + OPC is still under investigation in several running phase I-II [RTOG 3504 (NCT02764593)] and III trials [Keynote-412 (NCT03040999), CA209-9TM (NCT03349710), and CompARE trial (CRUK/13/026)] (Table 4).

**Table 4:** Running trials with immunotherapy in human papillomavirus related oropharyngeal carcinoma.

**Name study**	**Design**	**Inclusion TNM 7th**	**Inclusion TNM 8th (for p16+ OPC)**	**Smoking**	**Primary endpoint**
RTOG 3504 NCT02764593	4 arms: RT 70 Gy + weekly cisplatin + nivolumab 3-weekly cisplatin + nivolumab cetuximab + nivolumab nivolumab	OPC p16+: T1T2-Nb2N3; T4T3-N0N3, OPC p16-;OC, Larynx, HP:T1T2-N2aN3 or T3T4-Nx	OPC p16+: T1T2-N1N3 (excluding N1 with only ipsilateral LN); T3T4-Nx	OPC p16+: >10 PY or < 10 PY if T4 or N3	Dose limiting toxicity (DLT)
Keynote-412 NCT03040999	RT 70 Gy + 3-weekly cisplatin + Pembrolizumab vs. placebo	All locally advanced Head and neck squamous cell carcinoma's; independent of p16 status	All locally advanced Head and neck squamous cell carcinoma's; independent of p16 status	/	5Y-Event-free survival
CA209-9TM NCT03349710	Cisplatin eligible patient: RT 70 Gy + cisplatin + nivolumab vs. placebo Cisplatin ineligible patient: RT 70 Gy + Nivo vs. cetuximab	All locally advanced OPC, OC, HP, or larynx; independent of p16 status	All locally advanced OPC, OC, HP, or larynx; independent of p16 status	/	6Y-Event-free survival
CompARE CRUK/13/026	4 arms: RT 70 Gy (OTT: 7 weeks) + cisplatin RT 64 Gy (OTT: 5 weeks) + 3-weekly cisplatin RT 70 Gy (OTT: 7 weeks) + 3-weekly cisplatin + durvalumab surgery + RT + cisplatin	OPC p16+: T1T3-N2bN2c and all T4 or N3 OPC p16–: T1T4-N1N3 or T3T4-N0	OPC p16+: T1T3-N1N2 (excluding N1 with only ipsilateral LN); all T4 or N3	OPC p16+: T1T3-N2bN2c only included if more than 10 PY	Overall survival

*RT, radiotherapy; OPC, Oropharyngeal carcinoma; OC, oral cavity; HP, Hypopharynx; PY, smoking pack years; 5 (6)Y, up to 5 (6) years after end of treatment; OTT, Overall Treatment Time; LN, lymph nodes*.

HPV+ OPC are believed to benefit more from immunotherapy than HPV– OPC because of several factors. First, HPV + tumors express viral antigens which can be recognized as foreign by the patient's immune system leading to immune recognition and activation. Second, the preferred tumor location of HPV+ OPC is situated in the tonsils or base of tongue, two lymphoid tissues. This tumor location leads to the presence of a higher level of CD8+ and PD-1 tumor infiltrating lymphocytes and PDL-1 positive cells which may play a crucial role in the better response of HPV+ OPC to immunotherapy with PD-1 inhibitors such as nivolumab and pembrolizumab, and to cetuximab, as described above (23, 52).

### Proton therapy

Decreased treatment related toxicity by the use of proton therapy instead of photon therapy is still under investigation. The unique energy transfer of proton therapy, with the highest energy transfer at a specific depth inside the tissue, the Bragg peak, makes it possible to spear more healthy tissue located posterior of the tumor. A case matched analysis of 150 OPC, mainly HPV+, treated with proton therapy or photon therapy was performed showing comparable OS and PFS but reduced rate of feeding tube dependency and severe weight loss in patients treated with proton therapy (53). However, prospective multicenter randomized trials, such as the ongoing NCT01893307, are needed to validate such findings.

The proton RT technique is a quite expensive strategy and probably not beneficial for all patients. Therefore, some have proposed patient selection using a model based approach in which a proton and photon treatment plan is made for each patient and the expected reduction of toxicity with proton therapy is calculated. If the toxicity reduction is more than a pre-defined margin, the patient would undergo proton therapy (54). In future, this treatment and selection strategy need to be validated with incorporation of cost-effectiveness analysis as well as patient-reported outcomes.

## General conclusion

In the next decade, the optimal treatment approach for HPV+ OPC will be determined based on the results of several running trials. Sufficient follow up of all these studies is crucial in order to be confident that outcome is not compromised, since HPV+ disease shows a trend for later relapses than HPV– disease. We must emphasize that until the mature results of these trials are known the treatment of HPV+ OPC should remain unchanged and identical to the treatment of HPV– OPC. Furthermore, the result of the trials cannot be generalized to all HPV+ OPC. As described above, most trials have different inclusion criteria in terms of TNM stage and smoking pack years. In addition, there is no consensus on HPV detection method. Whether the future treatment for HPV+ OPC will consist of changes in concomitant therapy, reduction of RT dose, immunotherapy or proton therapy, patient selection will be pivotal.

## Author contributions

Each author has provided substantial contributions to warrant authorship. SD wrote the manuscript. All authors contributed to manuscript revision, read and approved the submitted version.

### Conflict of interest statement

The authors declare that the research was conducted in the absence of any commercial or financial relationships that could be construed as a potential conflict of interest.
